# From Pandemic Innovation to Platform Diversification: A Systematic Review of Clinical and Preclinical Development of Non–SARS-CoV-2 mRNA Vaccines

**DOI:** 10.3390/diseases14070230

**Published:** 2026-06-26

**Authors:** Shuaibu Abdullahi Hudu, Muhannad Alruwaili, Mohamed Soliman, Emad A. Morad, Ghusun M. Alhazimi, Abdulgafar Olayiwola Jimoh

**Affiliations:** 1 Center for Health Research, Northern Border University, Arar 91431, Saudi Arabia; 2 Department of Microbiology, Faculty of Medicine, Northern Border University, Arar 91431, Saudi Arabia; mohamed.sherif@nbu.edu.sa; 3 Department of Medical Laboratory Technology, Faculty of Applied Medical Sciences, Northern Border University, Arar 91431, Saudi Arabia; muhannad.alruwaili@nbu.edu.sa; 4 Department of Microbiology, Faculty of Medicine, Zagazig University, Zagazig 44519, Al-Sharqia Governorate, Egypt; emadmorad2010@gmail.com; 5 Department of Bacteriology Lab, Northern Border Regional Laboratory, Northern Borders Cluster, Arar 73211, Saudi Arabia; ghosonn1@gmail.com; 6 Department of Pharmacology and Therapeutics, Faculty of Basic Clinical Sciences, College of Health Sciences, Usmanu Danfodiyo University, Sokoto 840232, Nigeria; jimoh.abdulgafar@udusok.edu.ng

**Keywords:** mRNA vaccines, influenza vaccine, RSV, vaccine immunogenicity, cancer vaccines

## Abstract

**Background:** Messenger RNA (mRNA) vaccines have emerged as a versatile platform beyond SARS-CoV-2, with expanding applications in infectious diseases and oncology. However, comprehensive evidence synthesis of non-SARS-CoV-2 mRNA vaccines remains limited. **Methods:** This systematic review followed PRISMA 2020 guidelines and was registered in PROSPERO (CRD420261323500). MEDLINE, Embase, Web of Science, Scopus, ClinicalTrials.gov, and WHO ICTRP were systematically searched for studies published between 1 January 2000 and 28 February 2026. Eligible studies included phase I–III clinical trials and in vivo preclinical studies evaluating non-SARS-CoV-2 mRNA vaccines. Two reviewers independently screened studies, extracted data, and assessed risk of bias using RoB 2, ROBINS-I, and SYRCLE tools. Findings were synthesized narratively because of substantial heterogeneity. **Results:** A total of 40 studies met the eligibility criteria and were included in the review, comprising 20 clinical studies and 20 preclinical studies. Advanced clinical programs targeted influenza and respiratory syncytial virus (RSV), with phase III trials displaying seroconversion rates above 70% with good safety profiles. Preliminary phase I studies for HIV, cytomegalovirus, rabies, and personalized cancer mRNA vaccines showed promising humoral and cellular immune responses. Preclinical studies showed strong antibody and T-cell responses against malaria, tuberculosis, Group B Streptococcus, and Zika virus. Most adverse events were mild to moderate, while serious vaccine-related adverse events were uncommon. **Conclusions:** Non-SARS-CoV-2 mRNA vaccines demonstrate substantial translational potential across infectious disease and oncology applications. Although the vaccine candidates have demonstrated promising immunogenicity and safety, most are in the early stages of development. This highlights the need for large trials, long-term safety follow-up and better global representation.

## 1. Introduction

Messenger RNA (mRNA) vaccines represent one of the most significant technological advances in modern vaccinology. While the global rollout of mRNA vaccines during the coronavirus disease 2019 (COVID-19) pandemic made their safety, efficacy, and scalability apparent [1], the conceptual basis for mRNA vaccine technology was established decades earlier. Initial studies confirmed that exogenous in vitro–transcribed (IVT) mRNA could be delivered into host cells to specify antigen expression and evoke adaptive immune responses. Initial attempts at clinical translation were challenging due to poor mRNA stability, inefficient intracellular delivery, and potent activation of the innate immune system, which impaired antigen expression [2,3].

Later, critical technological developments enabled mRNA vaccines. Nucleoside modification, one of the major advances enabling mRNA vaccines, reduces innate immune recognition while preserving translational efficiency [4]. Meanwhile, the advent of lipid nanoparticle (LNP) delivery systems enabled efficient cytosolic delivery of mRNA and provided enhanced in vivo stability at the molecular level, leading to increased antigen expression and immunogenicity [5]. These advances laid the groundwork for clinically useful mRNA vaccines that can trigger both humoral and cellular immunity. The COVID-19 pandemic accelerated global investment in mRNA vaccine research, development, and manufacturing, as the successful use of these vaccines spurred their development, approval, and production.

However, mRNA vaccine research extends beyond the response to SARS-CoV-2 and has been under investigation for decades. Early proof-of-concept human data indicated that mRNA vaccines, and more specifically mRNA encoding pathogen-derived antigens (for example, rabies and influenza), are well tolerated and induce immune responses in humans, suggesting that this platform can produce protective antibody responses against infectious agents [5,6,7,8]. Since then, the scope of non-SARS-CoV-2 mRNA vaccine development has expanded dramatically, with candidates in development for respiratory syncytial virus (RSV), cytomegalovirus (CMV), human immunodeficiency virus (HIV), Zika virus, and personalized cancer neoantigen therapy [7,8,9]. The vaccine platforms identified in this review encompass conventional non-replicating mRNA vaccines, self-amplifying RNA (saRNA) constructs, and emerging circular RNA technologies. Conventional mRNA vaccines rely on direct intracellular translation of the encoded antigen, whereas saRNA vaccines incorporate replication machinery that amplifies antigen production within host cells. Circular RNA constructs offer enhanced molecular stability and prolonged protein expression, making them attractive candidates for future vaccine development.

Compared to traditional vaccine platforms, mRNA vaccines have many technological and biological differences. Unlike conventional vaccines, which require external antigen production, mRNA vaccines use the host cell apparatus and protein synthesis machinery to produce target antigens after delivery of the mRNA construct into the cell. This expression of endogenous antigens is displayed with MHC class I and class II pathways, leading to activation of CD4^+^ helper T cells, CD8^+^ cytotoxic T lymphocytes, and B-cell–mediated antibody responses [5]. Furthermore, mRNA vaccines offer a high degree of manufacturing flexibility, as the antigen sequence can be rapidly redesigned and synthesized without requiring cell culture or egg-based production. Such a modular design allows for rapid adaptation to newly emerging pathogens and could also enable personalized vaccine strategies, particularly in oncology [8,9].

Non-SARS-CoV-2 applications include the furthest advanced clinical programs to date, vaccines against influenza and RSV. Enhancing neutralizing antibody induction to improve the immunogenicity of RSV vaccines has been achieved through diverse structural vaccinology strategies, including stabilization of viral glycoproteins in prefusion conformations. Similarly, data from large Phase III trials in the elderly demonstrate strong HAI responses and promising signals of efficacy for quadrivalent mRNA influenza vaccines [7]. Notably, data from early-phase clinical trials evaluating neoantigen mRNA vaccines in cancer demonstrate that such vaccines can generate tumor-specific CD8^+^ T-cell responses that can be strengthened when combined with immune checkpoint inhibitors [9].

Despite these advances, several important questions remain about the ultimate clinical translation of non-SARS-CoV-2 mRNA vaccines. The evidence base is heterogeneous by disease target, and most programs are in early-phase development. The long-term durability of immune responses beyond 1 year is incompletely characterized, and comparative efficacy data are sparse for many pathogens. Furthermore, the majority of clinical trials have occurred in high-income countries, jeopardizing global representativeness and equity in the future use of mRNA vaccines.

With an expanding, rapidly maturing landscape of RNA vaccine development, a comprehensive synthesis of available evidence is needed to clarify our progress across disease targets and define major translational gaps. Therefore, the objective of this systematic review was to evaluate non-SARS-CoV-2 mRNA vaccines in clinical and preclinical studies with respect to their immunogenicity and efficacy, safety profiles, and translational development stages. The specific aims of this review were: (1) to characterize the distribution of target diseases and development stages; (2) to summarize immunogenicity and clinical efficacy outcomes stratified by vaccine platforms; (3) to assess reported safety and reactogenicity profiles; and (4) to investigate translational and geographical disparities in clinical pathway development. This review aims to provide a holistic perspective on the non-SARS-CoV-2 mRNA vaccine arena (which continues to evolve) by synthesizing existing evidence across infectious disease and oncology applications, with implications for future research, policy, and vaccine development for emerging uses.

## 2. Methodology

### 2.1. Study Design and Protocol Registration

The details of this systematic review were strictly adhered to the Preferred Reporting Items for Systematic Reviews and Meta-Analyses (PRISMA 2020) guidelines to increase transparency and reproducibility. The review protocol was prospectively registered in PROSPERO (CRD420261323500) and is publicly accessible through the PROSPERO database. The main aim of this systematic review was to systematically assess and collate clinical and preclinical data on immunogenicity, efficacy, safety, and translational advancement of non-SARS-CoV-2 mRNA vaccine candidates.

### 2.2. Eligibility Criteria

Inclusion criteria were predefined according to the Population, Intervention, Comparator, Outcomes, and Study Design (PICOS) framework. In clinical studies, participants of any age group enrolled in Phase I–III trials evaluating non-SARS-CoV-2 mRNA vaccines were eligible. Furthermore, we included in vivo preclinical animal studies to obtain evidence from experimental models for translational applications. These preclinical studies encompassed all animal models, including murine and non-human primate systems, to assess the immunogenicity and/or protective efficacy of mRNA vaccine candidates.

Eligible interventions included mRNA vaccine platforms targeting infectious diseases or cancers other than SARS-CoV-2. These platforms include traditional nucleoside-modified mRNA vaccines, saRNA constructs, and novel circular RNA vaccine technologies. Hence, studies evaluating mRNA vaccine candidates targeting the broad spectrum of infectious diseases and oncology indications were also deemed relevant for this review. Comparators were placebo controls, licensed comparator vaccines, or standard-of-care interventions where applicable. For studies without a comparator group (e.g., single-arm phase I clinical trials), to ensure preliminary immunogenicity and safety data generated during early vaccine development were captured, these studies were also made eligible if conducted in an exploratory or early-phase setting.

The primary outcome of concern was vaccine immunogenicity, as measured by humoral or cellular immune responses. These variables included seroconversion rates, geometric mean antibody titers, neutralizing antibody responses, and antigen-specific T-cell responses. Secondary outcomes also included clinical efficacy endpoints, such as laboratory-confirmed infection, disease incidence reduction, or recurrence-free survival in oncology studies. Other outcomes included vaccine safety and reactogenicity profiles, covering local and systemic adverse events, as well as the longevity of immune responses when extended follow-up data were available.

Eligible study designs included randomized controlled trials, non-randomized interventional studies, and observational follow-up studies of clinical trials. To capture early translational evidence, in vivo (preclinical) animal studies were also included. Studies evaluating SARS-CoV-2 mRNA vaccines were excluded to maintain the review’s focus on the expanding applications of mRNA vaccine platforms beyond COVID-19. Given the extensive literature already available on SARS-CoV-2 vaccines, their inclusion would have disproportionately dominated the evidence base and obscured developments in other disease targets. Narrative reviews, editorials, commentaries, and conference abstracts without primary outcome data were also excluded. Language and region restrictions were not applied. The review included studies published from 1 January 2000 to 28 February 2026.

### 2.3. Information Sources and Search Strategy

A systematic literature review identified studies regarding non-SARS-CoV-2 mRNA vaccines. Systematic searches were conducted in the following electronic databases: MEDLINE (through PubMed), Embase, Web of Science Core Collection, and Scopus. Clinical trial registries, including ClinicalTrials.gov and the World Health Organization International Clinical Trials Registry Platform (WHO ICTRP), were also searched to identify ongoing or unpublished studies. The search strategy combined controlled vocabulary terms, such as Medical Subject Headings (MeSH) and Emtree terms, with free-text keywords related to mRNA vaccine technologies and relevant disease targets. Search terms included combinations of “mRNA vaccine,” “messenger RNA vaccine,” “self-amplifying RNA,” “RNA vaccine,” and “circular RNA vaccine.” These were pairwise combined with (disease-specific) keywords representing major vaccine targets under investigation, including influenza, respiratory syncytial virus, HIV, cytomegalovirus, tuberculosis, malaria, rabies, and cancer or neoantigen vaccines. Pure Boolean operators and database-specific filters were used when appropriate to narrow the search without compromising sensitivity. To ensure the review centered specifically on applications not used for SARS-CoV-2, relevant SARS-CoV-2 and COVID-19 search terms were excluded. Supplementary Material provides the complete search strategies for all databases, including specific keywords, subject headings, and Boolean combinations. To maximize retrieval of relevant studies, searches were conducted across multiple bibliographic databases, clinical trial registries, and reference lists of eligible studies. Following peer review, the search results and study selection process were re-evaluated to confirm the inclusion of all eligible non-SARS-CoV-2 mRNA vaccine studies published during the predefined search period.

### 2.4. Eligibility Assessment

All records retrieved through the database searches were imported into reference management software, and duplicate entries were identified and removed. The deduplicated dataset was then imported into the Rayyan systematic review platform to support screening. We performed study selection in two consecutive steps. In the initial stage, titles and abstracts of all retrieved records were independently screened by pairs of reviewers against predefined eligibility criteria. Studies that appeared potentially relevant were forwarded to the second stage, where full-text articles were obtained and independently evaluated by the same reviewers for final eligibility.

Disagreements between reviewers at either screening stage were resolved through discussion and consensus. When consensus could not be reached, a third reviewer served as an adjudicator to resolve discrepancies. Inter-reviewer agreement during the pilot title and abstract screening phase was high (Cohen’s κ = 0.84), indicating almost perfect agreement between reviewers regarding study eligibility. Data pertaining to study selection (identification, screening, eligibility, and inclusion) were documented and reported in accordance with the PRISMA 2020 flow diagram.

### 2.5. Data Extraction

A standardized data extraction form was created and piloted on a subset of the included studies to ensure consistency, completeness, and clarity prior to full data extraction. Two reviewers independently extracted data from all eligible studies, and any discrepancies were resolved by discussion and consensus. Where necessary, extracted information was checked back to the original publications, and additional material was used to clarify missing or unclear data.

Data collected from clinical studies included author, year of publication, country or geographic region, study design, clinical trial phase, sample size, participant demographics, and duration of follow-up. Intervention-related variables included target disease, mRNA vaccine platform type (e.g., conventional mRNA, self-amplifying RNA, or circular RNA), antigen composition, dosage regimen, and delivery system formulation (e.g., lipid nanoparticles). Data on animal models, experimental design, immunization schedules, and pathogen challenge models were collected for preclinical studies.

The primary endpoints were immunogenicity measures, including rates of seroconversion, geometric mean antibody titers (GMTs), neutralizing antibody responses, and antigen-specific CD4+ and CD8+ T-cell responses. Secondary endpoints in oncology studies included clinical efficacy endpoints such as laboratory-confirmed infection, reduction in disease incidence, pathogen clearance, and recurrence-free survival. Safety and reactogenicity outcomes, including local and systemic adverse events, serious adverse events, and dose-limiting toxicities, were also recorded.

Outcome measures reported in the included studies included percent seroconversion, geometric mean titers, neutralizing antibody titers, estimates of vaccine efficacy, frequencies of adverse events, and measures of cellular immune responses. When outcome data were presented in different formats, findings were standardized descriptively to allow comparison across studies. Funding sources and declarations of conflicts of interest were also documented when available.

Two reviewers independently assessed the risk of bias using design-specific validated tools. For randomized controlled trials, the Cochrane Risk of Bias 2 (RoB 2) tool was used. This tool is used to assess multiple methodological domains, including random sequence generation, allocation concealment, blinding of participants and personnel, incomplete outcome data, selective reporting of results, and other biases. The Risk of Bias in Non-randomized Studies of Interventions (ROBINS-I) tool was used to assess the risk of bias in non-randomized interventional studies. This framework takes into account the risk of bias introduced by confounding, participant selection, classification of interventions, deviations from intended interventions, missing data, and outcome measurement and reporting.

Preclinical animal studies were designed using the SYRCLE Risk of Bias tool, a standard for assessing the quality of experimental animal research. Areas for which training was provided include sequence generation, allocation concealment, blinding of investigators and outcome assessors, incomplete outcome data (e.g., drop-out), and reporting bias. The overall evaluation across these domains determined whether each study had low, some concerns, or high risk of bias. During the risk-of-bias assessment, disagreements were resolved by discussion and consensus amongst reviewers. Risk-of-bias findings are presented as aggregate domain-level summaries for each study category.

A quantitative meta-analysis was not performed due to substantial heterogeneity across the included studies in study design, vaccine platforms, target diseases, populations, animal models, immunogenicity endpoints, and reported outcome measures. The findings were synthesized using a structured narrative approach in accordance with PRISMA 2020 recommendations for narrative synthesis.

We classified studies by major analytic categories, including target disease, vaccine platform type (e.g., conventional mRNA, self-amplifying RNA, and circular RNA), and stage of development (clinical versus preclinical). Where applicable, clinical studies were further stratified by trial phase. This allowed us to compare findings across different vaccine technologies and disease targets while preserving distinctions in methodological quality and translational maturity.

We provided descriptive summaries of the extracted outcome measures, including seroconversion rates, geometric mean titers (GMTs), neutralizing antibody responses, antigen-specific T-cell responses, vaccine efficacy estimates, and adverse event frequencies. We standardized the data descriptively to allow for comparison across studies that reported outcomes in different formats. We presented the results using structured tables and narrative summaries organized by vaccine indication and development stage.

The synthesis also included methodological quality assessments with the RoB 2, ROBINS-I, and SYRCLE tools to guide the interpretation of findings. Particular attention was given to identifying patterns in immunogenicity, safety, reactogenicity, durability of immune responses, and translational progression across vaccine platforms and disease categories. Owing to the limited number of comparable late-phase studies and marked methodological diversity, formal statistical pooling, subgroup meta-analysis, and meta-regression analyses were not considered appropriate.

### 2.6. Subgroup and Sensitivity Analyses

Full details of sources and methods were reported to ensure full transparency in how subgroup analyses were undertaken, wherever sufficient opportunity was identified in the available data to isolate specific elements of the studies reviewed that might account for differences in outcomes. Importantly, these analyses were stratified by relevant variables, including vaccine platform type (e.g., conventional mRNA, self-amplifying RNA, and novel circular RNA). Furthermore, for studies that were stratified into early- and late-phase clinical studies (i.e., early versus late phase trials) at the clinical development stage, we addressed and described these differences between cohorts to provide a better sense of the longitudinal evolution of efficacy & safety environments. The geographic spread of registered clinical trials was also examined by cross-referencing studies from high-income countries with those from low- and middle-income countries.

In addition to subgroup analyses, we conducted sensitivity analyses to assess the robustness and reliability of the study’s results. These were strict sensitivity analyses that directly assessed whether studies judged at high risk of bias were influential, thereby examining how this could affect the interpretation of results based on pooled data. Where the risk of bias identified in studies was considered relevant, the results of any analyses were re-evaluated after excluding studies deemed to be at high risk of bias, so that it could be explicitly determined whether and to what extent inclusion or exclusion affected the direction or strength of the synthesized evidence.

### 2.7. Assessment of Reporting Bias

We assessed the risk of reporting bias by comparing the results documented in existing entries of clinical trial registries and study protocols with those published in the corresponding published articles. This comparison was used to detect discrepancies between prespecified and reported outcomes that could suggest selective outcome reporting. We focused on primary immunogenicity endpoints, safety outcomes, and measures of efficacy.

Publication bias was assessed qualitatively during evidence synthesis by comparing the number of published late-phase clinical trials with smaller early-phase or preclinical studies. However, a formal quantitative assessment of publication bias using funnel plots or regression-based methods was not performed owing to substantial methodological heterogeneity, the predominance of narrative synthesis, and the limited number of comparable studies within individual disease categories.

The available evidence base was limited by potential reporting bias in unpublished or incompletely reported early-phase vaccine studies, and findings from exploratory clinical and preclinical studies were interpreted with caution, particularly when data from long-term follow-up or independent validation studies were unavailable.

### 2.8. Ethical Considerations

This systematic review synthesized evidence from a wide range of previously published studies and publicly available clinical trial registries. Crucially, as the study involved no new data collection from human participants or animal subjects, ethical approval and informed consent were not required, simplifying the ethics landscape.

### 2.9. Declaration of Generative AI and AI-Assisted Technologies in the Manuscript Preparation Process

During the preparation of this work, the authors used Grammarly Version 1.2.98), Quill Bot (Version 4.18.0), and ChatGPT (OpenAI, San Francisco, CA, USA, GPT-5.5) to enhance English grammar and figures. After using this tool, the author(s) reviewed and edited the content as needed, and they take full responsibility for the content of the published article.

## 3. Results

### 3.1. Study Selection

The systematic searches of bibliographic databases and clinical trial registries identified 3976 records. After removing 1470 duplicate records, 2506 records underwent title and abstract screening. Following initial screening, 113 studies were considered potentially eligible and underwent full-text review. Of these, 73 reports were excluded for failing to meet the predefined eligibility criteria. The two policy-oriented publications were retained solely for contextual discussion and were not considered eligible clinical or preclinical studies. Consequently, 40 studies met the inclusion criteria and were included in the final qualitative synthesis.

The main reasons for excluding full-text articles were: studies focusing on SARS-CoV-2 or COVID-19 vaccines; studies lacking primary immunogenicity or safety outcome data; review articles, editorials, and conference abstracts without sufficient original data; in vitro-only experimental studies; and studies evaluating non-mRNA vaccine platforms.

**Figure 1:**
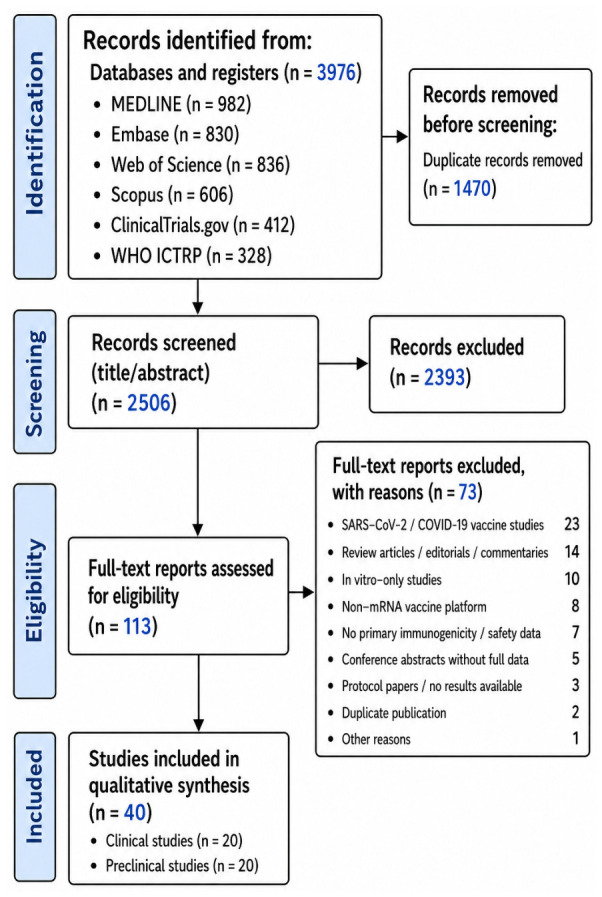
PRISMA 2020 flow diagram showing study identification, screening, eligibility assessment, and inclusion. Searches of bibliographic databases and clinical trial registries yielded 3976 records. After removal of 1470 duplicates, 2506 records were screened, 113 full-text reports were assessed for eligibility, and 40 studies (20 clinical and 20 preclinical) were included in the qualitative synthesis.

The included studies represented a broad range of applications of non-SARS-CoV-2 mRNA vaccines in infectious diseases and oncology. The complete study selection process, including identification, screening, eligibility assessment, and final inclusion, is summarized in the PRISMA 2020 flow diagram (Figure 1).

### 3.2. Study Characteristics

Forty studies (20 clinical and 20 preclinical studies) met the inclusion criteria and were included in the final qualitative synthesis. mRNA vaccine candidates are currently in clinical trials for a variety of infectious diseases and oncology indications. Influenza virus (*n* = 8 studies) and respiratory syncytial virus (RSV; *n* = 6 studies) were the most common pathogens studied, reflecting a relatively advanced state of development for mRNA vaccines targeting respiratory viruses. Large phase III trials have been conducted to evaluate the effectiveness of quadrivalent influenza and RSV mRNA vaccines [10]. This was followed by further clinical trials of mRNA vaccine candidates against HIV [11], CMV [12], and rabies virus [3]. These include early-stage clinical trials of personalized neoantigen mRNA vaccines for melanoma, further indicating that mRNA technology has expanded beyond simply preventing infectious diseases to treating cancers [9].

Sample sizes for clinical trials ranged widely based on the stage of development. Earlier phase studies, especially those investigating oncology vaccine candidates, often enrolled small cohorts of fewer than 20 patients, as these were exploratory evaluations of safety and immunogenicity [9]. In comparison, phase 3 studies of mRNA vaccines against respiratory viruses enrolled large cohorts of 30–40,000 individuals from multiple sites worldwide [10,13].

The 20 preclinical studies focused on experimental mRNA vaccine candidates against pathogens, including malaria, tuberculosis, Group B Streptococcus, and Zika virus, that were evaluated in murine or other animal models. Antigen expression, immunogenicity, and protective efficacy after pathogen challenge were evaluated in these investigations. Malaria mRNA vaccine candidates encoding Plasmodium falciparum circumsporozoite protein induced high anti-CSP IgG titers, robust interferon-γ–producing CD4^+^ T-cell responses, and sterile protection in a proportion of vaccinated animals following parasite challenge [14]. Likewise, mRNA vaccines targeting the Mycobacterium tuberculosis antigens Ag85B and ESAT-6 elicited strong T helper type 1 (Th1) immune responses, and tuberculosis mRNA vaccines elicited Th1-biased cellular immune responses characterized by increased antigen-specific CD4^+^ T-cell activation and significant reductions in pulmonary bacterial burden compared with unvaccinated controls [15,16]. Additionally, preclinical studies investigated multi-epitope mRNA vaccine constructs for Group B Streptococcus (GBS), demonstrating positive immunogenicity profiles in experimental systems [17].

Most of these vaccines employed lipid LNP delivery systems to enhance mRNA stability and intracellular delivery, while some studies explored saRNA constructs to increase antigen expression and subsequent immune activation. Most identified clinical trials were conducted in high-income regions, particularly North America and Europe, with relatively limited representation from low- and middle-income countries [10,11,12,13].

Most advanced clinical programs were for respiratory viruses, particularly influenza and RSV, with several phase III trials demonstrating robust immunogenicity and acceptable safety profiles [7,10,13,18,19]. Vaccine candidates for HIV, CMV, rabies, malaria, tuberculosis, and oncology indications were largely represented by early-phase clinical or preclinical studies [3,9,11,12,14,15,16,17,20]. Table 1 presents representative studies included in the review.

**Figure 2:**
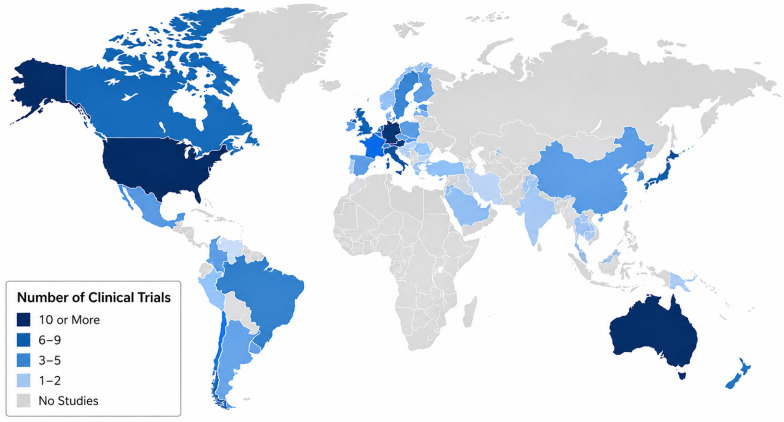
Global distribution of non-SARS-CoV-2 mRNA vaccine clinical trials. World map showing the geographic distribution of clinical trials evaluating non-SARS-CoV-2 mRNA vaccines identified in this systematic review. Figure created using BioRender.com.

Figure 2 shows the geographic distribution of clinical trials of non-SARS-CoV-2 mRNA vaccines identified in this review. Most of the registered clinical studies were conducted in high-income countries such as North America and Europe, reflecting the strong research infrastructure and manufacturing capacity in these regions [7,10,11,12,13,18,19]. This gap underscores the need to increase participation in global clinical trials, local manufacturing capacity, and technology transfer to ensure equitable access to future mRNA vaccine platforms. The United States, Australia, Germany, France, and the United Kingdom accounted for the highest concentration of registered clinical trials, whereas Africa and much of South America had limited representation.

### 3.3. Risk of Bias Assessment

Randomized controlled trials were assessed using the RoB 2 tool [22], non-randomized interventional studies using the Risk of Bias in ROBINS-I tool [23], and preclinical animal studies using the SYRCLE risk-of-bias tool [24]. Studies in phase III randomized controlled trials of influenza and RSV mRNA vaccines had a low overall risk of bias, with sound study designs that included proper allocation concealment, randomization procedures, outcome reporting, and blinding of participants/assessors [10,13]. In comparison, a number of early-phase clinical trials, especially those investigating potential HIV vaccines and personalized cancer vaccines, were rated as having a moderate risk of bias owing to open-label designs and small sample sizes, which may limit the generalizability of the findings [9,11].

Methodological diversity was greater in preclinical animal studies. Although some studies described appropriate randomization methods in their experimental designs, most did not clearly state whether allocation concealment or blinding of investigators or outcome assessors was used. As such, many preclinical studies were categorized as having moderate or unclear risk of bias, underscoring the need for improved reporting in experimental vaccine research. The risk-of-bias assessment across included studies is summarized in Table 2, which presents methodological domains evaluated using the RoB 2, ROBINS-I, and SYRCLE tools. Although multiple randomized clinical trials showed an overall low risk of bias, many early-phase clinical trials and preclinical studies were limited by moderate methodological concerns, mostly due to small sample sizes, open-label design, or incomplete reporting of randomization and blinding procedures. These limitations should be taken into consideration when discussing immunogenicity and safety results and point to a need for more stringent trial designs in mRNA vaccine research moving forward.

### 3.4. Immunogenicity Outcomes

Eight studies tested mRNA influenza vaccines, including large multicenter phase III trials. These vaccines induced robust humoral immune responses, with HAI seroconversion rates exceeding 70% across vaccine strains and geometric mean titers (GMTs) increasing by approximately 6- to 12-fold above baseline. In phase III studies, immunogenicity was non-inferior or superior to licensed egg-based influenza vaccines, with relative vaccine efficacy estimates favoring mRNA formulations in older adults [7,18]. In the phase III mRNA-1010 study, seroconversion rates exceeded 70% across vaccine strains, while GMT increases ranged from 6- to 12-fold above baseline. Relative vaccine efficacy estimates against laboratory-confirmed influenza ranged from approximately 26–27% compared with licensed standard-dose influenza vaccines. The six RSV mRNA vaccines evaluated targeted the prefusion F glycoprotein. High seroresponse rates were observed against both RSV-A and RSV-B strains, with neutralizing antibody titers increasing approximately 3- to 4-fold above baseline. Antibody responses remained detectable for up to 12 months following vaccination, supporting the durability of immune responses induced by RSV mRNA vaccines [10,19].

Immunogenicity signals were promising in early-phase clinical trials studying HIV, CMV, and rabies mRNA vaccines. Rabies mRNA vaccine candidates achieved 100% seroconversion by day 43 following a three-dose schedule, with virus-neutralizing antibody titers exceeding the WHO protective threshold in most participants [3]. Early phase HIV mRNA vaccine studies demonstrated detectable binding antibody responses in most vaccinated participants and measurable CD4^+^ and CD8^+^ T-cell responses following immunization, although the induction of broadly neutralizing antibodies remains a major challenge [8,11]. Glycoprotein B and the pentameric complex both showed robust neutralizing antibody responses in early-phase CMV mRNA vaccine trials [12]. Examples of personalized neoantigen mRNA vaccines in oncology applications include tumor-specific CD8^+^ T-cell responses induced by vaccination in patients with melanoma, as well as evidence for epitope spreading induced during combination therapy with immune checkpoint inhibitors [9].

### 3.5. Preclinical Efficacy Data

Twenty preclinical studies assessed the immunogenicity and protective efficacy of non-SARS-CoV-2 mRNA vaccine candidates in murine and other experimental animal models, with studies evaluating vaccines targeting infectious diseases, including malaria, tuberculosis, GBS, and Zika virus, and results predominantly related to antibody generation, cellular immune responses, pathogen clearance, and protection after experimental challenge.

Studies on saRNA and lipid nanoparticle-formulated mRNA vaccines against the Plasmodium falciparum circumsporozoite protein (CSP) induced strong humoral and cellular immune responses, including higher IgG titers and interferon-γ–producing CD4+ T-cell responses [14]. Vaccinated animals achieved sterile protection against the malaria parasite upon challenge in some murine challenge models. Similarly, mRNA vaccines encoding Mycobacterium tuberculosis antigens Ag85B and ESAT-6 induced Th1-biased immune responses with increased antigen-specific CD4+ T-cell activation and a significant reduction in the pulmonary bacterial burden after experimental infection [15,16].

Preclinical studies of Group B Streptococcus vaccine demonstrated strong opsonophagocytic antibody responses and protection of neonatal offspring in maternal immunization models [17]. Zika virus mRNA vaccine studies using the prM-E glycoprotein showed strong neutralizing antibody responses and reduced viremia after viral challenge. Table 3 summarizes the major preclinical efficacy, immune responses, and protective effects. Subgroup analyses showed that respiratory virus vaccine programs, particularly influenza and RSV vaccines, had more mature efficacy and safety data than oncology and intracellular pathogen vaccine programs, which are still largely in early-stage development. While respiratory-virus vaccines have advanced to large, multicenter phase III clinical trials, most mRNA vaccine candidates targeting tuberculosis, malaria, HIV, and cancer remain in exploratory preclinical or early-phase clinical evaluation.

Although most preclinical studies reported favorable immunogenicity and protection profiles, methodological heterogeneity was substantial across animal models, vaccine constructs, dosing schedules, and challenge systems. In addition, several studies incompletely reported experimental randomization, allocation concealment, and blinding procedures, which may affect reproducibility and interpretation of translational efficacy findings. Nevertheless, the collective evidence supports the capacity of mRNA vaccine platforms to induce coordinated humoral and cellular immune responses against complex intracellular pathogens and emerging infectious diseases. The interpretation of evidence strength was greater for late-phase influenza and RSV vaccine trials than for most early-phase and preclinical studies, due to differences in sample size, methodological quality, and follow-up duration.

### 3.6. Safety and Reactogenicity

The safety data from the individual vaccine candidates demonstrated broadly consistent safety profiles. Injection-site pain was the most commonly reported among local adverse events and affected a large number of participants. Across clinical studies, the majority of local and systemic adverse events were mild to moderate in severity. Injection-site pain was the most frequently reported local reaction, while fatigue, headache, myalgia, and low-grade fever were the most common systemic symptoms. Serious vaccine-related adverse events were uncommon, and no consistent safety concerns were identified across vaccine platforms [3,7,10,12,19]. A comparative overview of reactogenicity profiles and serious adverse events reported across clinical mRNA vaccine programs is presented in Table 4. Most of these adverse events were reported as mild to moderate in severity and generally resolved without medical intervention. On the contrary, serious adverse events were rarely reported and considered unrelated to vaccination, indicating a reassuring safety profile for the vaccines.

### 3.7. Sensitivity Analysis

Sensitivity analyses were conducted by re-evaluating the evidence after exclusion of studies judged to be at high risk of bias. The exclusion of these studies did not materially affect the overall interpretation of immunogenicity, efficacy, or safety outcomes across the included vaccine programs. The principal conclusions regarding the favorable immunogenicity of influenza and RSV mRNA vaccines, the promising early-phase findings for HIV, CMV, and oncology applications, and the encouraging preclinical efficacy data for malaria and tuberculosis remained unchanged. These findings suggest that the review’s overall conclusions were robust to potential methodological limitations identified during the risk-of-bias assessment.

## 4. Discussion

This systematic review summarizes existing clinical and preclinical evidence on the development of mRNA vaccines independent from SARS-CoV-2, demonstrating the diverse applications of this platform beyond simply pandemic response. The results of this study suggest that mRNA vaccine technology has progressed from an experimental construct to a versatile, increasingly validated platform suitable for the development of vaccines against multiple infectious diseases and oncology. Though concepts of mRNA vaccinology were defined over two decades ago [2], the rapid worldwide implementation of COVID-19 vaccines catalyzed extensive research, development, and investment in mRNA technologies. Consequently, mRNA vaccine development programs now include pathogens such as influenza, respiratory RSV, CMV, and HIV, as well as non-viral targets such as malaria and tuberculosis [14,15]. The rapidly evolving mRNA vaccine pipeline is substantially larger than represented in the present review because many candidates remain unpublished, are reported only in conference proceedings, or are currently undergoing preclinical and early-phase clinical evaluation.

The rapid development and global deployment of SARS-CoV-2 mRNA vaccines have provided important insights that have accelerated progress in the broader field of mRNA vaccinology. Clinical experience demonstrated that mRNA vaccines can induce robust neutralizing antibody responses alongside durable CD4^+^ and CD8^+^ T-cell immunity. Long-term follow-up studies further showed that booster immunization can restore waning antibody levels while maintaining cellular immune memory. These observations have informed the design of next-generation mRNA vaccines targeting influenza, RSV, CMV, HIV, and cancer-associated antigens. Furthermore, regulatory pathways, manufacturing platforms, and pharmacovigilance frameworks established during the COVID-19 pandemic have reduced barriers to clinical translation of new mRNA vaccine candidates.

Another important lesson from SARS-CoV-2 vaccine development concerns the critical role of lipid LNP delivery systems. Most clinical mRNA vaccines identified in this review utilized ionizable lipid-based LNP formulations similar to those employed in licensed COVID-19 vaccines. Although LNPs facilitate intracellular delivery and enhance antigen expression, differences in lipid composition may influence biodistribution, reactogenicity, and immunogenicity. Clinical experience with BNT162b2 and mRNA-1273 demonstrated that vaccine performance is determined not only by the encoded antigen but also by the delivery platform. Future comparative studies should evaluate how different LNP formulations affect vaccine efficacy, durability, and safety across disease targets.

Influenza and respiratory RSV vaccines are the most advanced non-SARS-CoV-2 mRNA vaccine programs among the targets discussed in this review. These vaccines have shown robust immunogenicity and an acceptable safety profile in large randomized clinical trials. HAI seroconversion rates of >70% have been reported across circulating influenza strains, and geometric mean antibody titers equivalent to or higher than those elicited by licensed egg-based vaccines [7,18]. These findings are biologically plausible, as the mRNA vaccine platform circumvents egg-adapted mutations that can modify antigenicity during the manufacturing of conventional influenza vaccines. Furthermore, mRNA constructs can be synthesized and modified relatively fast, allowing a vaccine to be adapted to a changing viral strain in a timely manner. Likewise, clinical trials of RSV mRNA vaccines targeting stabilized prefusion F glycoproteins have also shown strong neutralizing antibody responses and durable immunogenicity lasting at least 12 months [10,19]. Taken together, this data supports the ongoing development of mRNA platforms for respiratory viral pathogens.

Beyond this, the flexibility of mRNA vaccine technology for more complex viral pathogens such as HIV and CMV has been demonstrated in early-phase clinical trials. mRNA constructs encoding HIV envelope antigens have been shown to elicit humoral and cellular immune responses, including neutralizing antibodies and CD4^+^ and CD8^+^ antigen-specific T-cell responses [8]. Although broadly neutralizing antibodies remain one of the largest challenges in HIV development, the potential of mRNA platforms to rapidly reconfigure antigen constructs is facilitating iterative engagement with advances in structural biology and computational immunogen downstream design. Similarly, early-phase trials of CMV mRNA vaccine candidates elicited robust neutralizing antibody responses against both glycoprotein B and pentamer complex antigens, with antibody titers exceeding those observed following natural infection in some study cohorts [12].

In contrast to conventional prophylactic vaccines, therapeutic mRNA cancer vaccines are designed to induce tumor-specific immune responses against patient-specific or shared tumor antigens. Early clinical studies have demonstrated that personalized neoantigen mRNA vaccines can stimulate antigen-specific CD8^+^ T-cell responses in patients with melanoma and other solid tumors, supporting the feasibility of individualized cancer immunotherapy using mRNA technology [9]. These immune responses may be enhanced when mRNA vaccines are administered alongside immune checkpoint inhibitors, potentially improving antitumor activity by increasing antigen presentation, epitope spreading, and the expansion of tumor-reactive T-cell populations [9].

**Figure 3:**
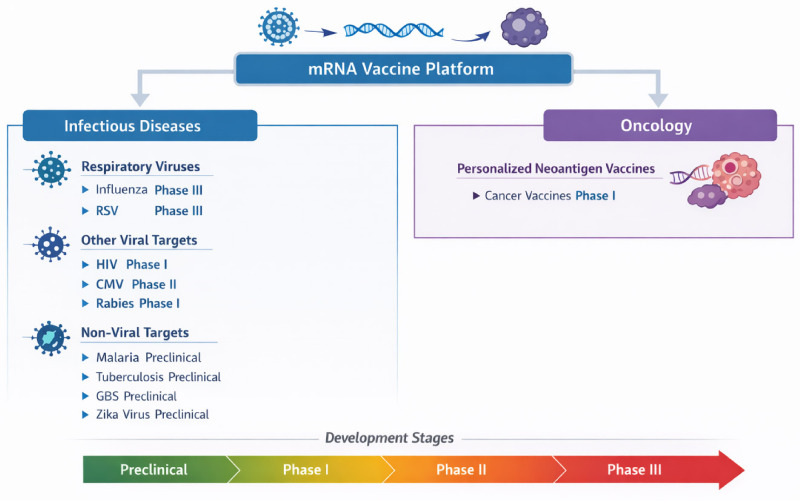
Landscape of Non-SARS-CoV-2 mRNA Vaccine Development Across Infectious Diseases and Oncology. The diagram summarizes major disease targets and development stages of mRNA vaccine candidates identified in this systematic review and highlights the expanding oncology pipeline represented by personalized neoantigen vaccine programs (created using BioRender.com).

The oncology applications identified in this review illustrate one of the most distinctive advantages of mRNA technology: the ability to rapidly design and manufacture vaccines tailored to the mutational profile of individual tumors. Unlike conventional cancer vaccines, which often target a limited set of predefined antigens, personalized mRNA vaccines can simultaneously incorporate multiple neoantigens, thereby addressing tumor heterogeneity and reducing the likelihood of immune escape. This flexibility has generated substantial interest in using mRNA vaccines as adjuncts to established immunotherapeutic approaches. The range of oncology and infectious disease targets currently under investigation is summarized in Figure 3.

Although clinical evidence remains limited compared with that for respiratory virus vaccine programs, the available data suggest that mRNA-based cancer vaccines can generate measurable cellular immune responses with acceptable safety profiles [9]. However, most oncology studies remain early-phase investigations with relatively small sample sizes and short follow-up periods. Consequently, additional randomized studies are required to determine whether the observed immunological responses translate into meaningful improvements in recurrence-free survival, progression-free survival, or overall survival. Taken together, the findings support continued development of mRNA-based cancer immunotherapies and highlight the growing role of mRNA technology beyond infectious disease prevention. As vaccine design, neoantigen prediction algorithms, and combination immunotherapy strategies continue to evolve, oncology is likely to remain one of the most important areas of future mRNA vaccine development.

Moreover, mRNA vaccines have been validated as a translational platform for pathogens that require potent cell-mediated immunity, as evidenced by the preclinical studies reviewed here. This is true of experimental mRNA vaccines encoding the malaria circumsporozoite protein, which induce robust antibody titers and interferon-γ-producing T-cell responses in mouse models and provide evidence of sterile protection upon parasite challenge [14]. Likewise, mRNA vaccines based on Mycobacterium tuberculosis antigens (Ag85B and ESAT-6) have been shown to induce a Th1-polarised immune response whilst reducing pulmonary bacterial burden following infection of mice [16]. These insights indicate that mRNA vaccine platforms may be especially well-suited for intracellular pathogens in which coordinated humoral and cellular immunity is required for protection. Despite methodological heterogeneity across animal studies and insufficient reporting of experiments, these findings underscore the need for greater standardization in preclinical vaccine development.

Although mRNA sequence design is a critical determinant of vaccine performance, the delivery system also contributes substantially to immunogenicity and reactogenicity. Most studies included in this review employed lipid LNP-based delivery systems. However, LNP compositions differed among vaccine developers in ionizable lipids, phospholipids, cholesterol content, and polyethylene glycol (PEG)-lipid components. Experience from licensed SARS-CoV-2 mRNA vaccines suggests that differences in LNP composition may influence antigen expression, biodistribution, tolerability, and the frequency of local and systemic adverse reactions. Future studies should provide more detailed reporting of LNP formulations to facilitate comparison across vaccine platforms.

The safety profiles reported in the studies reviewed were generally consistent with prior experience from SARS-CoV-2 mRNA vaccines. The most frequently reported adverse events comprised transient local injection-site reactions, together with mild systemic symptoms (fatigue, headache, and myalgia). Serious adverse events were uncommon, and most were deemed unrelated to vaccination. While rare inflammatory complications, including myocarditis, have been observed following large-scale administration of SARS-CoV-2 mRNA vaccines, non-SARS-CoV-2 vaccine trials thus far do not appear to show consistent safety signals [19]. Nonetheless, ongoing long-term safety monitoring will be critical as these vaccines move into larger clinical trials and eventually to widespread rollout. Post-marketing surveillance of SARS-CoV-2 mRNA vaccines has provided valuable information regarding vaccine safety. While most adverse effects are transient and self-limiting, including injection-site pain, fatigue, headache, and myalgia, rare adverse events such as myocarditis, pericarditis, and selected immune-mediated conditions have been reported. Importantly, these events remain uncommon, and extensive pharmacovigilance data continue to support a favorable benefit-risk profile for mRNA vaccine technology. Although mRNA vaccines have demonstrated favorable overall safety profiles, post-marketing surveillance of SARS-CoV-2 vaccines identified rare adverse events, including myocarditis, pericarditis, and isolated autoimmune manifestations. While such events have not been consistently observed in the non-SARS-CoV-2 mRNA vaccine programs reviewed here, long-term pharmacovigilance and active safety monitoring remain essential as these platforms are introduced into larger and more diverse populations.

Another significant finding of this review relates to the persistence of immune responses elicited by mRNA vaccines. Although persistently high, neutralizing antibody responses post influenza and RSV vaccination have been observed to last at least a year [10,19], long-term response kinetics remain poorly defined for many vaccine targets beyond this timeframe. Identifying reliable correlates of protection and establishing optimal booster schedules will thus be critical to future vaccine development and implementation. Most of the identified clinical trials were conducted in high-income countries, particularly in North America and Europe, with few conducted in low- and middle-income countries. This discrepancy could restrict the applicability of clinical data and increase public health concerns about equitable access to novel mRNA vaccines. These disparities can be partially addressed by expanding global manufacturing capacity, creating regional mRNA production hubs, and encouraging technology transfer initiatives [25,26].

These findings from this systematic review clearly demonstrate the momentum for mRNA vaccines shifting away from being merely a pandemic-fueled technology into a novel, adaptable platform for broad-spectrum vaccinology. Although the success of COVID-19 vaccines has proven that large-scale deployment of mRNA vaccines is possible, the growing pipeline of mRNA vaccines targeting influenza, RSV, HIV, CMV, malaria, tuberculosis, and cancer signals that mRNA technology has entered a new phase of translational development. Most vaccine candidates are in early clinical stages, underscoring the need for large, multicenter, randomized trials and long-term safety surveillance. Concurrently, addressing geographic disparities in clinical research and manufacturing capacity will be crucial to ensuring equitable access to future mRNA vaccines. Ongoing innovations in antigen design, delivery systems, and self-amplifying RNA technologies will likely further improve immunogenicity and scalability, with mRNA platforms becoming a cornerstone of next-generation vaccine development.

Several vaccine programs have advanced beyond the developmental stages represented in the included publications. For example, RSV mRNA vaccines have completed pivotal phase III studies and entered regulatory review processes in several jurisdictions. Similarly, combination influenza–COVID-19 mRNA vaccines have progressed through late-stage clinical evaluation. Conversely, some programs, including certain CMV vaccine candidates, have encountered challenges in achieving predefined efficacy endpoints. These developments illustrate the dynamic nature of the mRNA vaccine landscape and should be considered when interpreting findings from published studies. The oncology pipeline for mRNA vaccines continues to expand rapidly, with ongoing research focusing on personalized neoantigen vaccines, shared tumor-associated antigens, and combination approaches involving immune checkpoint blockade [9]. Advances in tumor sequencing technologies, bioinformatics-based neoantigen prediction, and mRNA manufacturing platforms are expected to improve vaccine precision and scalability. These developments may broaden the applicability of mRNA vaccines across a wider range of malignancies and contribute to more individualized approaches to cancer treatment. The growing diversification of mRNA vaccine applications beyond infectious diseases is illustrated in Figure 3.

Since several studies included in this review were completed, important regulatory developments have occurred. The RSV mRNA vaccine mRNA-1345 has received regulatory approval in several jurisdictions for the prevention of RSV-associated lower respiratory tract disease in older adults, demonstrating successful translation of the platform beyond SARS-CoV-2. Conversely, despite encouraging phase II immunogenicity findings, the CMV vaccine mRNA-1647 did not meet its primary efficacy endpoint in phase III. These examples highlight both the promise and challenges associated with clinical translation of mRNA vaccine technologies and underscore the importance of continuously updating evidence syntheses in rapidly evolving fields.

## 5. Limitations

Several limitations should be considered when interpreting the findings of this review. The field of mRNA vaccine research is evolving rapidly, with numerous vaccine candidates progressing through preclinical and clinical development. Consequently, some relevant studies may not have been available in the indexed literature or public trial registries at the time the searches were conducted. In particular, ongoing investigations, recently completed trials, and studies reported only through preliminary communications may not be fully represented.

Another limitation concerns the considerable diversity among the included studies in disease targets, vaccine constructs, study populations, outcome measures, and developmental stages. This variability limited direct comparisons across studies and prevented quantitative pooling of results. Furthermore, much of the available evidence originated from early-phase clinical trials and experimental animal studies, which are primarily designed to evaluate safety, immunogenicity, and proof-of-concept outcomes rather than definitive clinical effectiveness.

The review may also underrepresent certain therapeutic areas, including some oncology and non-oncology vaccine programs, owing to incomplete reporting, publication lag, or insufficient primary outcome data for inclusion. Finally, the predominance of studies conducted in high-income settings may restrict the generalizability of the findings to regions with different healthcare infrastructures, disease burdens, and research capacities. Future updates to this review, incorporating emerging evidence from ongoing trials and broader geographic representation, will provide a more comprehensive assessment of the expanding non-SARS-CoV-2 mRNA vaccine landscape.

## 6. Conclusions

Non-SARS-CoV-2 mRNA vaccines have evolved from early experimental platforms to sophisticated clinical pipelines for various infectious and oncologic indications. Influenza and RSV vaccines are the most advanced programs, with extensive randomized trials showing significant immunogenicity and favorable safety profiles. In contrast, despite promising immunological readouts and limited efficacy data, HIV, CMV, malaria, and tuberculosis, as well as personalized oncology applications, still reside in earlier development phases. While safety findings across multiple indications are reassuring, longer-term durability and the detection of rare adverse events will necessitate further evaluation. The future success of mRNA vaccine technology will depend on the conduct of rigorous late-phase trials, harmonized regulatory pathways, ongoing pharmacovigilance, and equitable scaling up of global manufacturing capacity. Maintaining strict methodological quality and developing internationally representative studies will be necessary to fully leverage this platform’s translational potential.

## Figures and Tables

**Table 1:** Representative Clinical and Preclinical Studies Included in the Review of Non-SARS-CoV-2 mRNA Vaccines.

Author/Year	Country/Region	Target Disease	Vaccine Platform	Study Type	Development Stage	Sample Size/Model	Main Outcomes
Alberer et al., 2017 [3]	Germany	Rabies	Conventional mRNA	Clinical trial	Phase I	Healthy adults (*n* = 101)	100% seroconversion; favorable safety
Feldman et al., 2019 [6]	USA	Influenza (H10N8/H7N9)	Conventional mRNA	Clinical trial	Phase I	Adults (*n* = 50)	Strong antibody responses; acceptable reactogenicity
Soens et al., 2025 [7]	Multinational	Seasonal influenza	mRNA-1010	Clinical trial	Phase III	Adults ≥ 50 years	>70% seroconversion; favorable safety
Kandinov et al., 2025 [18]	Multinational	Seasonal influenza	Conventional mRNA	Clinical trial	Phase III	Large adult cohort	Robust HAI titers; efficacy signals
Goswami et al., 2025 [19]	Multinational	RSV	mRNA-1345	Clinical trial	Phase III	Adults ≥ 50 years	Durable neutralizing antibodies; acceptable safety
ClinicalTrials.gov NCT05127434 [10]	Multinational	RSV	mRNA-1345	Clinical trial	Phase III	Adults ≥ 60 years	Safety and efficacy evaluation
ClinicalTrials.gov NCT06557785 [11]	USA	HIV	mRNA-gp160	Clinical trial	Phase I	Healthy adults	Immunogenicity and safety
ClinicalTrials.gov NCT04232280 [12]	USA	CMV	mRNA-1647	Clinical trial	Phase II	Healthy adults	Strong neutralizing antibodies
Sahin et al., 2017 [9]	Germany	Melanoma	Personalized neoantigen mRNA	Clinical trial	Early phase	Melanoma patients	Tumor-specific CD8+ T-cell responses
Wu et al., 2025 [14]	USA	Malaria	saRNA-LNP	Preclinical	Experimental	Murine model	Sterile protection; strong IgG responses
Lukeman et al., 2025 [15]	Australia	Tuberculosis	LNP-mRNA	Preclinical	Experimental	Murine model	Th1-biased immunity; reduced bacterial load
Touray, 2025 [16]	Experimental	Tuberculosis	mRNA nanovaccine	Preclinical	Experimental	Mouse model	Robust cellular immunity
Barazesh et al., 2024 [17]	Iran	Group B Streptococcus	Multi-epitope mRNA	Preclinical	Experimental	Murine maternal model	Protective neonatal immunity
McLellan et al., 2013 [21]	USA	RSV	Prefusion F design	Preclinical	Experimental	Animal models	Enhanced neutralizing responses
ClinicalTrials.gov NCT05581641 [20]	Multinational	Malaria	RNA-based vaccine	Clinical trial	Early phase	Healthy adults	Safety and immune responses

RSV = respiratory syncytial virus; CMV = cytomegalovirus; HIV = human immunodeficiency virus; HAI = hemagglutination inhibition; saRNA = self-amplifying RNA; LNP = lipid nanoparticle.

**Table 2:** Methodological quality assessment of the 40 included studies evaluating non-SARS-CoV-2 mRNA vaccines. Study-level assessment records were used during the review process; however, the aggregated presentation was retained because it more clearly summarizes methodological quality across study categories.

Study Type	Assessment Tool	Bias Domain	Low Risk (*n*)	Some Concerns/Moderate Risk (*n*)	High Risk (*n*)
Randomized Controlled Trials (*n* = 8)	RoB 2	Randomization process	7	1	0
		Deviations from intended interventions	7	1	0
		Missing outcome data	8	0	0
		Measurement of outcomes	7	1	0
		Selection of reported results	7	1	0
Non-Randomized Clinical Studies (*n* = 14)	ROBINS-I	Confounding	5	9	0
		Participant selection	6	8	0
		Classification of interventions	10	4	0
		Outcome measurement	11	3	0
		Selective reporting	12	2	0
Preclinical Animal Studies (*n* = 20)	SYRCLE	Sequence generation	6	9	5
		Allocation concealment	4	10	6
		Blinding of investigators/outcome assessors	3	9	8
		Incomplete outcome data	15	4	1
		Selective outcome reporting	16	3	1

RoB 2 = Cochrane Risk of Bias 2 tool for randomized controlled trials. ROBINS-I = Risk of Bias in Non-randomized Studies of Interventions tool. SYRCLE = Systematic Review Center for Laboratory Animal Experimentation, a risk-of-bias tool used for preclinical animal studies. Risk-of-bias judgments were classified as low risk, some concerns/moderate risk, or high risk based on the criteria of each assessment framework. Low risk signifies minimal methodological limitations; some concerns/moderate risk indicates possible bias that could affect interpretation; high risk reflects significant methodological flaws that may undermine internal validity. Risk-of-bias domains included randomization, allocation concealment, blinding, outcome measurement, incomplete outcome data, and selective reporting. These assessments were performed independently by two reviewers, with disagreements resolved through discussion and consensus.

**Table 3:** Key Preclinical Efficacy and Immune Outcomes for Non-Viral Targets.

Target	Antigen	Animal Model	Immune Response	Protective Outcome
Malaria	Circumsporozoite protein (CSP)	Murine challenge model	High IgG titers; IFN-γ–secreting CD4+ T cells	Sterile protection in subset of mice
Tuberculosis	Ag85B/ESAT-6 constructs	Murine aerosol infection model	Th1-biased CD4+ responses	Reduced pulmonary bacterial load
Group B Streptococcus	Surface protein constructs	Murine maternal model	High opsonophagocytic antibody titers	Protection of neonatal pups
Zika (preclinical)	prM-E glycoprotein	Mouse model	Neutralizing antibodies	Protection from viremia

CSP = circumsporozoite protein (Plasmodium falciparum antigen); Ag85B = antigen 85B (Mycobacterium tuberculosis secreted protein); ESAT-6 = early secretory antigenic target-6 (Mycobacterium tuberculosis antigen); IFN-γ = interferon gamma; Th1 = T helper type 1 immune response; murine model = experimental study conducted in mice; sterile protection = complete absence of detectable infection following pathogen challenge; bacterial load = quantity of viable bacteria recovered from infected tissue.

**Table 4:** Safety and Reactogenicity Profiles for Clinical mRNA Vaccine Candidates.

Vaccine Target	Key Immunogenicity Outcome	Safety Outcome
Influenza	Seroconversion > 70%; GMT rise 6–12-fold	Mostly mild-moderate AEs
RSV	Neutralizing antibody increase 3–4-fold	Comparable to placebo
Rabies	100% seroconversion by day 43	No vaccine-related SAEs
HIV	Detectable CD4+/CD8+ responses	Acceptable early-phase safety
CMV	Strong neutralizing antibody responses	Rare serious AEs
Malaria	Sterile protection in animal models	Preclinical
Tuberculosis	Reduced bacterial burden	Preclinical

AE = adverse event; SAE = serious adverse event; Reactogenicity = expected transient inflammatory response following vaccination; Placebo = inactive comparator administered in controlled trials; Comparator vaccine = licensed vaccine used as active control.

## Data Availability

The data supporting the findings of this study are available within the article and its Supplementary Materials.

## Supplementary Materials

The following supporting information can be downloaded at: https://www.mdpi.com/article/10.3390/diseases14070230/s1, File S1: Complete Search Strategies.
