# Supplementary material for: From Pandemic Innovation to Platform Diversification: A Systematic Review of Clinical and Preclinical Development of Non–SARS-CoV-2 mRNA Vaccines
Source: Diseases. 2026 Jun 26;14(7):230. doi: 10.3390/diseases14070230 (PMC13409428; doi:10.3390/diseases14070230)
Supplement: Supplementary file 1 [file diseases-14-00230-s001.zip › diseases-4345743-supplementary.pdf]

Supplementary Material File S1. Complete Search Strategies

Search period: 1 January 2000 to 28 February 2026

Final search date: 28 February 2026

1. MEDLINE (PubMed)

Date searched: 28 February 2026

("mRNA vaccine"[Title/Abstract]
OR "messenger RNA vaccine"[Title/Abstract]
OR "RNA vaccine"[Title/Abstract]
OR "self-amplifying RNA"[Title/Abstract]
OR "saRNA"[Title/Abstract]
OR "circular RNA vaccine"[Title/Abstract]
OR "circRNA vaccine"[Title/Abstract])
AND (influenza
OR "respiratory syncytial virus"
OR RSV
OR HIV
OR "human immunodeficiency virus"
OR cytomegalovirus
OR CMV
OR rabies
OR malaria
OR tuberculosis
OR cancer
OR oncology
OR neoantigen
OR melanoma
OR "Group B Streptococcus"
OR GBS
OR Zika)
NOT
("SARS-CoV-2"
OR COVID-19
OR coronavirus)

2. Embase

Date searched: 28 February 2026

('mRNA vaccine':ti,ab

OR 'messenger RNA vaccine':ti,ab
OR 'RNA vaccine':ti,ab
OR 'self amplifying RNA':ti,ab
OR 'saRNA':ti,ab
OR 'circular RNA vaccine':ti,ab
OR 'circRNA vaccine':ti,ab)
AND
('influenza'/exp
OR 'respiratory syncytial virus infection'/exp
OR 'human immunodeficiency virus infection'/exp
OR 'cytomegalovirus infection'/exp
OR 'rabies'/exp
OR 'malaria'/exp
OR 'tuberculosis'/exp
OR 'cancer vaccine'/exp
OR 'melanoma'/exp
OR 'neoantigen'/exp
OR 'group b streptococcus infection'/exp
OR 'zika virus infection'/exp)
NOT
('severe acute respiratory syndrome coronavirus 2'/exp
OR 'coronavirus disease 2019'/exp)

3. Scopus

TITLE-ABS-KEY(
"mRNA vaccine"
OR "messenger RNA vaccine"
OR "RNA vaccine"
OR "self-amplifying RNA"
OR saRNA
OR "circular RNA vaccine"
OR "circRNA vaccine")
AND
TITLE-ABS-KEY(
influenza
OR RSV
OR "respiratory syncytial virus"
OR HIV
OR cytomegalovirus
OR CMV
OR rabies
OR malaria
OR tuberculosis
OR cancer
OR oncology

OR neoantigen
OR melanoma
OR "Group B Streptococcus"
OR Zika)
AND NOT TITLE-ABS-KEY(
"SARS-CoV-2"
OR COVID-19)

4. Web of Science Core Collection

Date searched: 28 February 2026

TS=("mRNA vaccine"
OR "messenger RNA vaccine"
OR "RNA vaccine"
OR "self-amplifying RNA"
OR saRNA
OR "circular RNA vaccine"
OR "circRNA vaccine")
AND(influenza
OR RSV
OR "respiratory syncytial virus"
OR HIV
OR cytomegalovirus
OR CMV
OR rabies
OR malaria
OR tuberculosis
OR cancer
OR oncology
OR neoantigen
OR melanoma
OR "Group B Streptococcus"
OR Zika)
NOT
("SARS-CoV-2"
OR COVID-19)

5. ClinicalTrials.gov

Date searched: 28 February 2026

Searches were performed using combinations of:

mRNA vaccine

combined with:

influenza
RSV
HIV
CMV
rabies
malaria
tuberculosis
cancer
melanoma
neoantigen

Completed, active, and recruiting studies were screened for eligibility.

6. WHO International Clinical Trials Registry Platform (ICTRP)

Date searched: 28 February 2026

Searches were conducted using:

mRNA vaccine

combined with:

influenza
RSV
HIV
CMV
rabies
malaria
tuberculosis
cancer
neoantigen

All retrieved records were screened for eligibility.

7. bioRxiv

Date searched: 28 February 2026

Search terms:

"mRNA vaccine"
"messenger RNA vaccine"
"self-amplifying RNA"
"circular RNA vaccine"

combined with disease-specific keywords:

influenza
RSV
HIV
CMV
malaria
tuberculosis
cancer
melanoma

8. medRxiv

Date searched: 28 February 2026

Search terms:

"mRNA vaccine"
"messenger RNA vaccine"
"self-amplifying RNA"
"circular RNA vaccine"

combined with:

influenza
RSV
HIV
CMV
rabies
malaria
tuberculosis
cancer
melanoma
neoantigen
Study Selection and Documentation

All retrieved records were imported into reference management software to remove duplicates. Deduplicated records were subsequently uploaded into Rayyan for title/abstract screening and full-text review. Search results, screening decisions, exclusions, and final study selection were documented in accordance with PRISMA 2020 guidelines. The complete study selection process is presented in Figure 1 of the manuscript.
